# HH Domain of Alzheimer’s Disease Aβ Provides Structural Basis for Neuronal Binding in PC12 and Mouse Cortical/Hippocampal Neurons

**DOI:** 10.1371/journal.pone.0008813

**Published:** 2010-01-21

**Authors:** Joseph F. Poduslo, Emily J. Gilles, Muthu Ramakrishnan, Kyle G. Howell, Thomas M. Wengenack, Geoffry L. Curran, Karunya K. Kandimalla

**Affiliations:** 1 Molecular Neurobiology Laboratory, Departments of Neurology, Neurobiology, and Biochemistry/Molecular Biology, Mayo Clinic College of Medicine, Rochester, Minnesota, United States of America; 2 Department of Pharmacy and Pharmacology, Florida Agricultural and Mechanical University, Tallahassee, Florida, United States of America; Mental Health Research Institute of Victoria, Australia

## Abstract

A key question in understanding AD is whether extracellular Aβ deposition of parenchymal amyloid plaques or intraneuronal Aβ accumulation initiates the AD process. Amyloid precursor protein (APP) is endocytosed from the cell surface into endosomes where it is cleaved to produce soluble Aβ which is then released into the brain interstitial fluid. Intraneuronal Aβ accumulation is hypothesized to predominate from the neuronal uptake of this soluble extracellular Aβ rather than from ER/Golgi processing of APP. We demonstrate that substitution of the two adjacent histidine residues of Aβ40 results in a significant decrease in its binding with PC12 cells and mouse cortical/hippocampal neurons. These substitutions also result in a dramatic enhancement of both thioflavin-T positive fibril formation and binding to preformed Aβ fibrils while maintaining its plaque-binding ability in AD transgenic mice. Hence, alteration of the histidine domain of Aβ prevented neuronal binding and drove Aβ to enhanced fibril formation and subsequent amyloid plaque deposition - a potential mechanism for removing toxic species of Aβ. Substitution or even masking of these Aβ histidine residues might provide a new therapeutic direction for minimizing neuronal uptake and subsequent neuronal degeneration and maximizing targeting to amyloid plaques.

## Introduction

Extracellular accumulation of Aβ in different soluble monomeric or polymeric forms and its extracellular aggregation leading to plaque formation within the hippocampus and cortex is one of the hallmark pathologies of AD [1]. It has been estimated that 70% of the interstitial fluid Aβ arises from endocytosis-associated mechanisms in neurons which is dependent upon synaptic activity rather than from ER/Golgi processing of APP [2]. During AD pathogenesis, the Aβ can exist as soluble Aβ monomers or oligomers or aggregate into insoluble species, including protofibrils and fibrils, which ultimately result in amyloid plaque formation. Intraneuronal accumulation of Aβ may arise from the uptake of this soluble pool of Aβ into neurons that disrupts its normal function ultimately leading to apoptosis [3] and oxidative injury [4] that seems to occur even before the formation of senile plaques and neurofibrillary tangles. Wirths et al. [5] have recently reviewed biochemical, neuropathological, and genetic information that indicates Aβ accumulation in neurons precedes its formation as amyloid plaques in the extracellular space and have hypothesized that intraneuronal Aβ accumulation is the first step of a fatal cascade of events leading to neurodegeneration in AD. Numerous reports have supported this viewpoint (also reviewed by Echeverria and Cvello [6], Tseng et al. [7], and Gouras et al. [8]), and include Mochizuki et al. [9], who have reported that cells that were immunoreactive for Aβ42 are localized around amyloid plaques in sporadic AD cases and Gouras [10] who demonstrated intraneuronal AB staining was most evident in brain regions that show the first signs of plaque accumulation, such as entorhinal cortex and hippocampus. A good example of the neuropathological consequences of intraneuronal Aβ can be found in presenilin mutation bearing AD transgenic mice which show extensive intraneuronal Aβ accumulation and subsequent neuronal death without any amyloid plaque formation in the brain [11]. Aβ-burdened neurons undergo lysis and the aggregated Aβ may be the nidus for extracellular plaque formation as it is released into the extracellular space [12].

Recently, Kandimalla et al. [13] reported that fluorescein-labeled Aβ40 was selectively accumulated in a subpopulation of cortical and hippocampal neurons and in PC12 cells via non-saturable, energy independent, and non-endocytotic pathways through a mechanism that most likely results in biophysical interaction with the neuronal membrane. This finding challenges the conventional belief that Aβ proteins are internalized via receptor-mediated endocytosis. A large portion of the internalized Aβ40 was located outside of the endosomal or lysosomal compartment, and it was hypothesized that the protein could accumulate in the neuroplasm without degradation where it could subsequently aggregate to form fibrils, which ultimately results in the degeneration of the neuron. Understanding the mechanism of neuronal uptake of Aβ is an important direction to take as it might lead to potential therapeutic targets that would minimize this uptake and the subsequent neurodegeneration that leads to the pathogenesis of AD.

In this study, we hypothesize that a specific domain of Aβ protein provides a structural basis for its neuronal binding, which ultimately triggers its internalization. It has been suggested that Aβ-microglial interactions occur through membrane-associated heparin sulfate interactions with the HHQK domain of Aβ [14]. This same cluster of basic amino acids is also known as a domain with high-binding affinity for heparin sulfate. These authors have suggested that HHQK-like agents may offer a specific therapy to block plaque-induced microgliosis. Epitope mapping studies have revealed that positively charged amino acid clusters with common epitopes of Aβ (HHQKL) and APO-E (LRKRL) are regions that are known to be heparin-binding domains [15]. These authors have substituted Aβ residues with glycine (GGQGL) and found that they failed to compete with the cellular uptake of APO-E enriched βVLDL. They suggest that these observations indicate that Aβ and APO-E are taken up into cells by a common pathway involving heparin sulfate proteoglycans. Diaz et al. [16] have observed that small peptides of Aβ that have replacements of histidine 13 and 14 by alanine or lysine block the Aβ ion channel activity. Tickler et al. [17] demonstrated that methylation in the imidazole side chains of all three histidine residues on Aβ resulted in a four-fold increase in H_2_O_2_ generation. Despite the higher levels of H_2_O_2_, the modified peptides were not neurotoxic. These modifications both altered Cu binding and inhibited peptide/cell surface membrane interaction. In similar studies by the same group, Smith et al. [18] determined that copper-mediated Aβ toxicity was associated with an intermolecular bridge involving histidines 6, 13, and 14 of Aβ. Methylation of these histidine residues was shown to be non-toxic in cell culture systems, because these chemical modifications inhibited the interaction between the peptide and the cell surface membrane. Interestingly, three amino acid differences exist in a comparison of human Aβ peptide with the rodent Aβ peptide (Arg-5→Gly; Tyr-10→Phe and His-13→Arg). Despite these three amino acid substitutions, no difference was observed in the ability of either rodent or human peptides to form fibrils in aqueous buffer [19]-[21], yet rats and mice do not innately develop age-associated amyloid pathology [22]. Replacement of the His-13 for Arg in rodent Aβ disrupts the metal coordination site which results in the rodent peptide having less ability to zinc-induced aggregation *in vitro* [23]-[25]. In addition, the rodent form of Aβ that has His13 mutated to an Arg has been shown to be non-toxic [26]. Indeed platinum-based compounds that target histidine residues on Aβ inhibit Aβ toxicity [27]. All of these studies point to a critical role of histidine residues at positions 13 and 14 on Aβ protein. As a result, we decided to evaluate the effects of H13,14 substitution of Aβ40 on its ability to affect neuronal binding in a culture model of differentiated PC12 cells and in cortical and hippocampal neurons in WT mouse brain slices. These *in vitro* systems have been well characterized and used by numerous investigators as models to study neuronal physiology [28]-[31]. Because of the well recognized involvement of Aβ in forming fibrils and amyloid plaques, we also evaluated the effects of H13,14 substitution of Aβ40 on its subsequent ability to form fibrils, bind to Aβ fibrils, and label amyloid plaques.

## Methods

### 2.1 Peptides

Human Aβ1-40, with the sequence DAEFRHDSGYEVHHQKLVFFAEDVGSNKGAIIGLMVGGVV, Aβ1-40 H13G with the sequence DAEFRHDSGYEVGHQKLVFFAEDVGSNKGAIIGLMVGGVV, Aβ1-40 H13,14G with sequence DAEFRHDSGYEVGGQKLVFFAEDVGSNKGAIIGLMVGGVV, Aβ1-40 H13R with sequence DAEFRHDSGYEVRHQKLVFFAEDVGSNKGAIIGLMVGGVV, and Aβ1-40 R5G, Y10F, H13R (rodent Aβ1-40) with sequence DAEFGHDSGFEVRHQKLVFFAEDVGSNKGAIIGLMVGGVV were synthesized with or without Ahx (Fmoc-6-aminohexanoic acid) attached to the N-terminal on a CEM Liberty (Mathews, NC) peptide synthesizer using HBTU activation and the manufacturer’s suggested synthesis protocols. The starting resin was Val-NovaSyn TGA (Novabiochem EMD Biosciences, San Diego, CA). For fluoresceinated peptides, after completion of the synthesis and final Fmoc deprotection, NHS-fluorescein, [5-(and 6)-carboxyfluorescein, succinimidyl ester] (Pierce) was added to the N-terminal Ahx residue by dissolving fluorescein in 2 ml of DMSO/8 ml DMF and reacting the fluorescein solution with the peptide-resin, which had been washed previously with DIEA/DCM. The coupling of fluorescein was allowed to proceed overnight at RT. The peptides were then cleaved from the resin support using 5% crystalline phenol, 5% water, 2.5% triisopropylsilane, and 87.5% TFA for two hours at RT. The peptide was purified by reverse-phase HPLC on a Jupiter C18 column (250 mm×21.2 mm, Phenomenex Corp) using a gradient system of 0.1% aqueous TFA containing 80% acetonitrile/water/0.1% TFA. The calculated mass weight was 4329 amu for Aβ1-40; 4249 amu for Aβ1-40 H13G; 4169 amu for Aβ1-40 H13,14G; 4348 amu for Aβ1-40 H13R; and 4233 amu for Aβ1-40 R5G, Y10F, H13R as confirmed by electrospray ionization mass spectrometry (Thermoelectron Surveyor MSQ).

### 2.2 Cell Culture

PC12 cells were maintained in DMEM supplemented with 10 mM HEPES (pH 7.4), 10% fetal bovine serum, 4 mM L-glutamine, penicillin (200 U/mL) and streptomycin (200 µg/mL) in 5% CO_2_ at 37°C. Cells were plated at 1.5×10^4^ cells per well in uncoated 6-well Falcon plates (Becton Dickinson Labware, Franklin Lakes, NJ) and allowed to differentiate for 6-7 days in medium supplemented with 100 ng/mL nerve growth factor (Harlan Biosciences).

### 2.3 PC12 Interactions with F-Aβ Derivatives

PC12 cells were equilibrated with 37°C HBSS (Hanks balanced salt solution) for 30 minutes prior to the start of an experiment. After equilibration, cells were incubated with a freshly prepared 20 µM F-Aβ solution in HBSS containing 10 µM AlexFluor-633 transferrin for 20 minutes. Cells were removed from each well with either non-enzymatic cell dissociation medium or trypsin. Each sample was centrifuged at 1000×g for 5 minutes and resuspended in PBS. Unfixed cells were analyzed on a FACSCalibur (Becton-Dickinson, San Jose, CA) flow cytometer. Argon ion laser light (λ = 488 nm) was used to excite fluorescein, and emission fluorescence was detected with a 585±21-nm filter. Helium neon laser light (λ = 635 nm) was used to excite the Alexafluor-633 transferrin, and emission fluorescence was detected by 647 nm. Forward-angle scatter, side scatter, and geometric mean fluorescent intensities were recorded from 20,000 cells and analyzed using Cell Quest (Becton-Dickinson) and WinMDI software. Only cells were analyzed above a forward-angle scatter threshold that distinguishes healthy cells. The time from the initiation of incubation with freshly prepared F-Aβ40 to FACS analysis was less than 30 minutes which was well below the time required for fibril formation; hence, these studies were restricted to monomeric Aβ. The geometric means and the coefficients of variance were determined using WinMDI version 2.8 software. Statistical significance between geometric means was determined by the Mann-Whitney Rank Sum Test using GraphPad InStat software.

### 2.4 Brain Slice Binding of F-Aβ40 and F-Aβ H13,14G

Wild-type (WT; B6/SJL strain) mice at 4 months of age were used in procedures approved by the Mayo Clinic Institutional Animal Care and Use Committee in strict accordance with the National Institutes of Health Guide for the Care and Use of Laboratory Animals and. All mice were sacrificed with Nembutal (200 mg/kg; Ovation Pharmaceuticals, Inc., Deerfield, IL) and immediately perfused via the aorta with 12ml of 4°C Krebs-Ringer Bicarbonate Buffer (KRB) pH = 7.3 (Sigma-Aldrich, St. Louis, MO). The animals were decapitated and their brains were carefully removed from the cranial cavity. Brains were embedded in 2% agar and cut coronally, by Vibratome (Lancer, St. Louis, MO) into 500 µm-thick slices. Agar was removed; tissue slices containing cortex and hippocampus were cut into hemisections and placed in oxygenated (95% O_2_/5% CO_2_) KRB at 4°C. Following equilibration in KRB, each brain slice was incubated for five minutes at room temperature in either KRB alone, KRB containing FITC (50 µg/ml) or KRB containing fluorescein-labeled peptide (F-Aβ40 or F-Aβ40 H13,14G; 50 µg/ml; all freshly prepared). Brain slices were acid stripped to remove extracellular peptides in a 4°C acidified KRB (pH = 5.0) containing no peptide for 5 minutes. Tissue slices were briefly washed four times in KRB (pH = 7.3), mounted on a coverslip (ThermoFisher Scientific, Inc., Waltham, MA) and imaged with constant gain using a Zeiss LSM 510 laser confocal microscope (excitation = 488 nm; emission = 520 nm, Carl Zeiss, Inc., Thornwood, NY). The images were analyzed using AxioVision digital imaging software (Carl Zeiss, Inc., Thornwood, NY). The mean intensity of all fluorescent cells in the images for each brain region for each peptide was quantified in an unbiased manner using a semi-automated image analysis macro. Statistical analysis of F-Aβ40 and F-Aβ40 H13,14G mean intensities in the cortex and hippocampus of 4 month old WT mice was performed by two-way ANOVA using GraphPad Prism 4 software (GraphPad Software, Inc., La Jolla, CA).

### 2.5 Thioflavin-T Solution

ThT was prepared as described by Khurana et al. [32]. Briefly, approximately 3 mg of ThT (Fluka) was dissolved in 1 mL water. This solution was further diluted in PBS and filtered through a 0.22-µm syringe filter (Millipore). The concentration of this stock solution was measured at 416 nm (extinction coefficient: 26,620 M^-1^ cm^-1^). The solution was stored at 4°C covered in foil and used up to one week to make assay solutions.

### 2.6 Fluorescence Assay for Fibril Formation

The fluorescence excitation spectrum of ThT shifts from 340 to 450 when interacting with β-sheet structures. Fluorescence signals (excitation, 430 nm; emission, 485 nm) reflected the amount of amyloid fibrils formed. Peptides were dissolved in nanopure H_2_O and briefly sonicated (two 10-sec pulses) before PBS was added to bring the concentration to 200 µM. Peptides were filtered through 0.22-µm syringe filters (Millipore). Aliquots of each peptide were added to ThT and PBS to give a final peptide concentration of 20 µM and 5 µM ThT in each well of a 384-well plate (Corning). The plate was incubated at 37°C for three days in a GENios plate reader (Tecan, Maennedorf, Switzerland), with a three minute shaking period and fluorescence reading every 20 minutes. The formation of fibrils was assessed by the maximum fluorescence signal at the end of 2.5 days and the presence of fibrils was confirmed by TEM.

### 2.7 Fibril Formation Kinetics of Aβ40 Derivatives

The ThT fluorescence signal of various Aβ40 derivatives against time was fitted to a kinetic model that is routinely used to analyze protein aggregation kinetic data (Morris et al.). The model is based on the Finke-Watzky 2-step mechanism. The first step assumes slow continuous nucleation followed by a second step of fast autocatalytic surface growth.
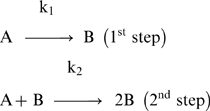



This model suggests that the fibril formation is initiated by the aggregation of monomers (A) to form nuclei (B) at the rate of k_1_. These aggregates are extended by the addition of monomers to form large fibrils (2B) at the rate of k_2_. The nonlinear curve fitting was achieved using WinNonlin^®^ Professional, version 5.2 (Pharsight, Mountain View, CA). Parameters such as the rate constants k_1_ and k_2_, and the amount of 2B formed were estimated for each Aβ40 derivative.

### 2.8 TEM

Carbon-coated Formvar 300 mesh grids (Electron Microscopy Sciences) were spotted with 3 µL of sample taken from the 384-well plate immediately after the fibril formation experiment was stopped. The negative stain was performed using 2% uranyl acetate. The grids were stained for 5 minutes and rinsed with nanopure H_2_O three times. The samples were observed using a transmission electron microscope (JEOL Ltd., Tokyo, Japan).

### 2.9 Surface Plasmon Resonance

Surface Plasmon Resonance (SPR) analyses were performed at 25^o^C using Biacore 3000 optical biosensors with research grade CM5 chips (Biacore, Uppsala, Sweden). Aβ40 fibrils were grown in PBS buffer pH 7.4 at 37^o^C under constant shaking at 250 rpm for 48 hrs. The fibrils were sonicated in a bath sonicator for 3 min, reconstituted in 10 mM sodium acetate pH 4.0, and about 80 RU (Relative Units) of Aβ40 fibrils were immobilized using carbodiimide chemistry to the CM5 chip surface. The binding sensograms were recorded following the injection of freshly prepared peptides over the immobilized surface for 5 min at a flow rate of 30 µl/min. The dissociation profile was monitored for about 15 min, and then the surface was regenerated with two short 8 sec injections of 50 mM NaOH. Extensive validation studies were performed to ensure that Aβ fibrils immobilized on the chip retained their binding characteristics even after the regeneration procedure. Kinetic data analysis was performed using Biaevaluation software (Version 3.2) provided by Biacore Inc., Uppsala, Sweden.

### 2.10 Labeling of Amyloid Plaques in APP, PS1 Mouse Brain Sections In Vitro with Radioiodinated Aβ40, Aβ40 H13G, Aβ40 H13R, or Aβ40 H13,14G

HPLC-purified ^125^I-Aβ40, ^125^I-Aβ40 H13G, ^125^I-Aβ40 H13R, ^125^I-Aβ40 H13,14G or buffer was incubated *in vitro* with unfixed cryosections (15 µm) of brain from a 20-month old APP, PS1 AD transgenic mouse using the same procedure used previously [33]. The rodent Aβ was not evaluated because the peptide lacks a tyrosine amino acid for radioiodination. Briefly, the sections were incubated for 3 hrs with 100 pM radioiodinated peptide or alone in 250 µl of TBS (50 mM Tris HCl, 138 mM sodium chloride, pH 7.0) containing 0.1% BSA, 0.6 mg/ml magnesium chloride, 0.04 mg/ml bacitracin, 0.002 mg/ml chymostatin, and 0.004 mg/ml leupeptin. After rinsing and air drying overnight at 4°C., the sections were fixed in formalin for 5 min and then underwent immunoperoxidase histochemistry (IH) for amyloid using an anti-Aβ monoclonal mouse antibody (4G8, 1∶1000, Signet Laboratories, Dedham, MA). Next, the sections were dipped in an autoradiographic emulsion (Type NTB-3, Kodak, Rochester, NY) for direct comparison of ^125^I-peptide labeling to anti-Aβ IH. The slides were dipped in emulsion, exposed for various durations, and developed according to the instructions. The sections were dehydrated with successive changes of ethanol and xylene and then coverslipped.

## Results

### 3.1 Binding of F-Aβ40 and F-Aβ40H13,14G to PC12 Cells

**Figure 1:**
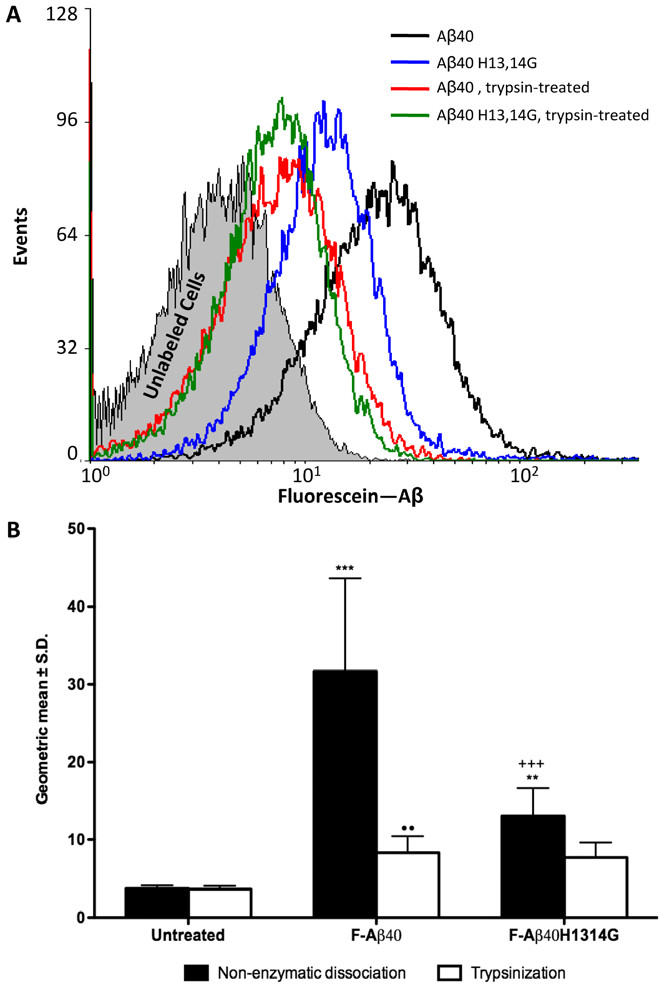
Binding of F-Aβ40 and F-Aβ40H13,14G to PC12 cells as quantified flow cytometry. PC12 cells were incubated with fluorescein-labeled Aβ peptides and cells were harvested using either trypsin or non-enzymatic cell dissociation medium. (A) Histograms of cellular fluorescence obtained from flow cytometry analysis. (B) Geometric means averaged from at least 8 experiments were found to be significantly different where indicated, as determined by one-way ANOVA (p<0.0001). ***p<0.001 = Untreated cells versus the cells treated with F-Aβ40H13,14G; ^+++^p<0.001 = The cells treated with F-Aβ40 versus the cells treated with F-Aβ40H13,14G; ^••^p<0.01 = F-Aβ40 treated cells harvested by non-enzymatic dissociation versus the cells harvested by trypsinization.

Rat pheochromocytoma (PC12) cells were differentiated with nerve growth factor-containing medium and used as a neuronal cell culture model to assess the binding of of fluorescein-labeled Aβ40 (F-Aβ40) and Aβ40 H13,14G (F-Aβ40 H13,14G). Shifts in histograms of cellular fluorescence obtained by flow cytometry analysis indicated mostly membrane-association and/or cellular uptake of F-Aβ with differentiated PC12 cells (Figure 1A). In addition to being incubated with F-Aβ, transferrin conjugated to AlexaFluor-633 was included as a marker of endocytosis and healthy, viable cells (data not shown) [34]. Comparison of the geometric means of these histograms revealed significant differences in the ability of F-Aβ40 and F-Aβ40H13,14G to bind to PC12 cells (Figure 1B). Unlabeled cells show the level of background autofluorescence. Cells treated with non-enzymatic cell dissociation medium display cellular fluorescence from both internalized and membrane-associated peptide. Treatment of cells with trypsin removes the membrane-associated peptide and represents F-Aβ internalization only. Trypsin-treatment decreases the fluorescence in cells treated with F-Aβ and F-Aβ40H13,14G to the background levels, indicating that the cellular internalization of these proteins is insignificant at the shorter incubation times used in this study. Modification of the H13,14 domain significantly decreases cellular binding compared to native Aβ40 (Figure 1B, black bars). Following the trypsin treatment, the fluorescence in cells incubated with either peptide returned to background levels observed in untreated cells (Figure 1B, white bars). The cells treated F-Aβ40 H13,14G did not show any significant difference before and after trypsin treatment.

### 3.2 Neuronal Interaction with Aβ Derivatives in Brain Slices

**Figure 2:**
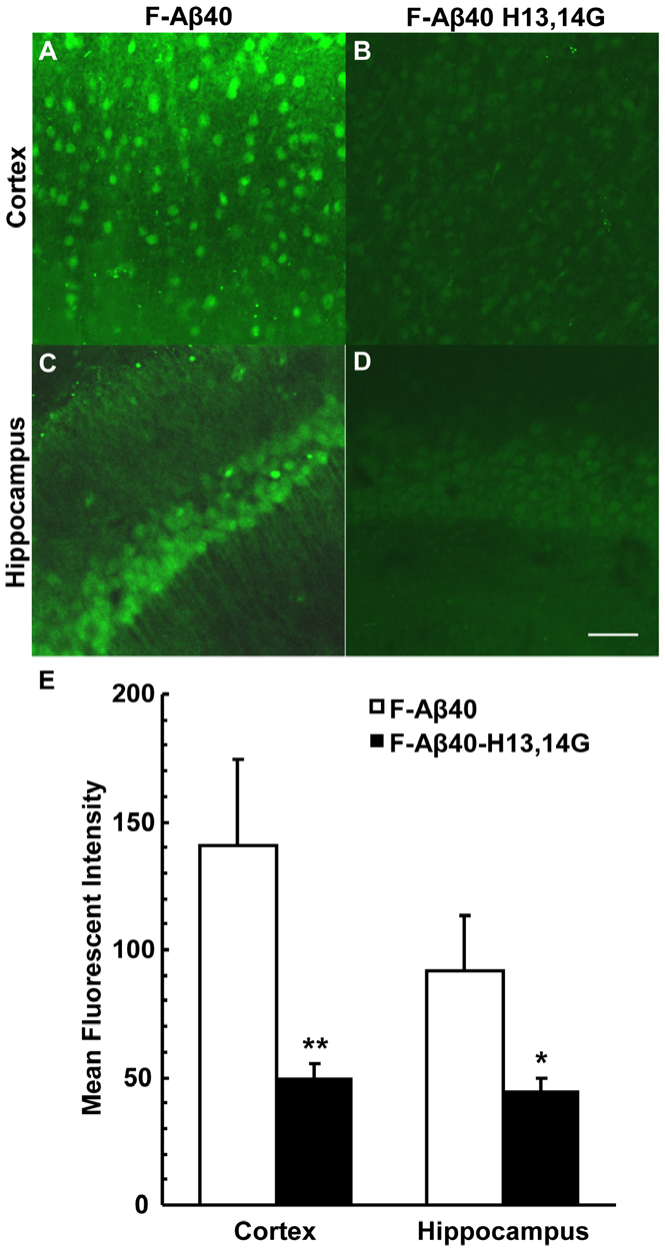
In vitro binding of F-Aβ40 and F-Aβ40 H13,14G in neurons of 4 month old WT brain slices. (A) Cortex incubated with F-Aβ40. (B) Cortex incubated with F-Aβ40 H13,14G. (C) Hippocampus incubated with F-Aβ40 (D) Hippocampus incubated with F-Aβ40 H13,14G. Scale bar equals 50 µm. (E) Mean fluorescent intensity of cortical and hippocampal neurons from brain slices incubated with F-Aβ40 or F-Aβ40 H13,14G. Analysis of Variance (ANOVA) [F(1,8) = 33.73; p<0.001] followed by Bonferroni post-hoc multiple comparisons: Cortex - **p<0.01; Hippocampus - *p<0.05.

Brain slices from 4 month old WT mice were incubated with F-Aβ40 or F-Aβ40 H13,14G to assess cellular interaction (Figure 2). The binding of the Aβ peptides appeared to be selective for cortical neurons based on the pyramidal morphology of the fluorescent cells. A comparison of mean fluorescent intensity of cortical neurons in these WT mice show a significant decrease (p<0.01) in F-Aβ40 H13,14G binding when compared to F-Aβ40 (Figure 2A, B, and E). Following F-Aβ40 incubation, cortical neurons had a mean fluorescence intensity of 141±34 (mean ± SD), whereas brain slices incubated with F-Aβ40 H13,14G had a mean fluorescence intensity of 50±6 which represents a 65% decrease in binding. To verify neuronal binding, the hippocampus was also evaluated. F-Aβ40 binding in the hippocampus was observed only in the pyramidal cells of the stratum pyamidale. The distinct apical dendrites of the CA1 pyramidal cells are also fluorescent and can be observed in Fig. 2C as long parallel fibers projecting from the cell bodies in the lower right of the micrograph. Although we did not perform any neuron-specific staining ourselves, the pattern of fluorescence we observed in the CA1 hippocampal subfield matches the neuron-specific immunohistochemical staining as observed by Miya et al. [35]. A significant decrease (p<0.05) in F-Aβ40 H13,14G binding by hippocampal neurons when compared to F-Aβ40 was also observed (Figure 2C, D and E). Hippocampal neurons following F-Aβ40 incubation had a mean fluorescence intensity of 92±22, whereas brain slices incubated with F-Aβ40 H13,14G had a mean fluorescence intensity of 44±6 which represents a 52% decrease in binding. These brain slice experiments were performed a total of three times with the same results. These data suggest modifying F-Aβ40 to F-Aβ40 H13,14G by substituting glycine for histidine at domains 13 and 14 significantly decreases neuronal binding in the cortex and hippocampus.

### 3.3 Fibril Formation of Aβ Peptides

**Figure 3:**
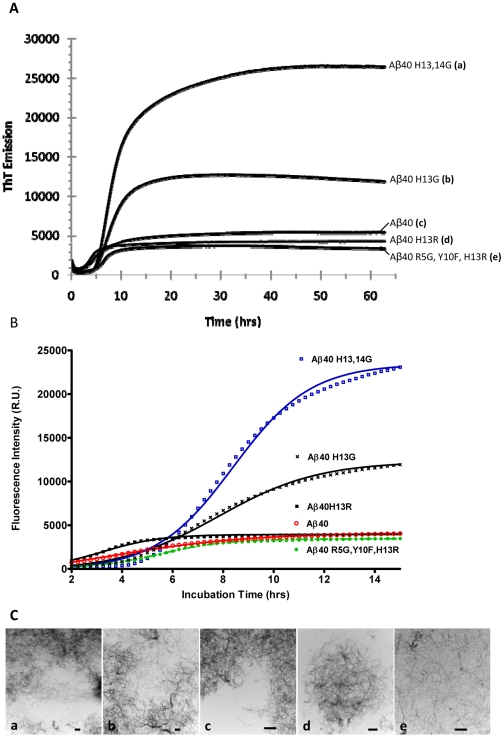
Fibril formation of Aβ derivatives. (A) Thioflavin T signal versus time of amyloid β peptides. Peptide samples, at 20 µM and containing thioflavin T, were excited at 430 nm and the emissions at 485 nm were monitored over 2.5 days at 37°C with periodic shaking. For each peptide, n = 18. (B) Thio-T fluorescence representing the fibril formation kinetics of various Aβ40 derivatives fitted to the Finke-Watzsky 2-step aggregation model [36]. The observed data has been indicated by various symbols. The predicted data obtained through curve-fitting is presented as solid lines. (C) TEM confirmation of the presence of amyloid fibrils formed by the corresponding amyloid β peptide described in panel A (a: Aβ40 H13,14G; b: Aβ40 H13G; c: Aβ40; d: Aβ40 H13R; e: Aβ40 R5G, Y10F, H13R). The sample was collected at the end of the fibril formation experiment and applied to a grid and negatively stained with uranyl acetate. Scale bar equals 1 µm.

The fibril formation kinetics of the Aβ40 derivatives were determined based on the changes in ThT fluorescence intensity with time. The modification of the Aβ40 histidine 13 to arginine (H13R) slightly increased the fibril formation rate relative to Aβ40 while modification of histidine 13 to glycine (H13G) substantially increased the fibril formation rate (Figure 3A). However, it is interesting to note that the lag time with H13G modification increased remarkably compared to the lag time with the H13R modification (Table 1A). More strikingly, modifying the entire histidine 13,14 domain to glycine dramatically enhanced the rate of fibril formation, which is again associated with a significant increase in the lag time. The rodent form of Aβ (Aβ40 R5G, Y10F, H13R) did not exhibit significant differences in the rate of fibril formation compared to the human Aβ40, yet the lag time was greater for the rodent form (Table 1A). Due to the unusual increases in the lag time associated with increases in the rates of fibril formation, the kinetics were studied in greater detail by fitting the data to a model that was developed to describe the aggregation of neurological proteins [36] (Figure 3B). This model is based on the Finke-Watzky mechanism of nucleation, described by the rate constant k_1_, followed by autocatalytic surface growth, described by the rate constant k_2_.

**Table 1:** Fibril Formation Kinetics of Aβ40 Derivatives.

A) Lag time and slope obtained from thioflavin T fluorescence graphs		
Peptide	Lag time, hrs	Slope, RU/hr
Aβ40 H13,14G	4.92	3501±80.6
Aβ40H13R	1.10	911±21.0
Aβ40 H13G	3.70	1507±27.3
Aβ40	0.88	539±5.0
Aβ40 R5G, Y10F, H13R	2.97	676±9.6

B) Kinetic parameters obtained by fitting experimental data to the protein aggregation model. [36]			
Mutant	k_1_ (x10^3^), hr^-1^	k_2_ (x10^3^), hr^-1^	Max. fluorescence intensity (R.U.)
Aβ40 H13,14G	2.726±2.7	0.028±0.001	23438±332
Aβ40 H13R	0.001±0.0008	0.218±0.038	3713±263
Aβ40 H13G	9.3±7.6	0.041±0.002	12292±210
Aβ40	79.3±14.1	0.066±0.007	4143±110
Aβ40 R5G, Y10F, H13R	0.0001±0.0001	0.219±0.012	3397±55

k_1_: rate of nucleation.

k_2_: rate of fibril growth.

The rate constants estimated by the model were highly significant based on the student t-test. The rate of nucleation of Aβ40H13R and the rodent Aβ40 are substantially lower (∼1000 fold) than that of human Aβ40; whereas, the rate of surface growth is significantly greater for these Aβ derivatives than human Aβ40 (Table 1B). The rate constants k_1_ and k_2_ obtained by fitting aggregation model to H13G fibril formation data were about 1/10^th^ and 2/3^rd^, respectively of the rate constants for native human Aβ40. But the ThT fluorescence intensity was significantly greater than that of native human Aβ40 (Table 1B). These trends became even more pronounced with the H13,14G modifications. Moreover, the aggregation model did not fit the Aβ40H13,14G data as well as it did the other proteins, which suggests the possibility that Aβ40 proteins with H13G and/or H14G modifications likely follow a mechanism different from Finke-Watzky mechanism of nucleation and autocatalytic surface growth. The presence of fibrils associated with these interesting kinetic trends was confirmed by TEM (Figure 3C).

### 3.4 Surface Plasmon Resonance Analysis of Aβ Derivatives to Immobilized Aβ40 Fibrils

**Figure 4:**
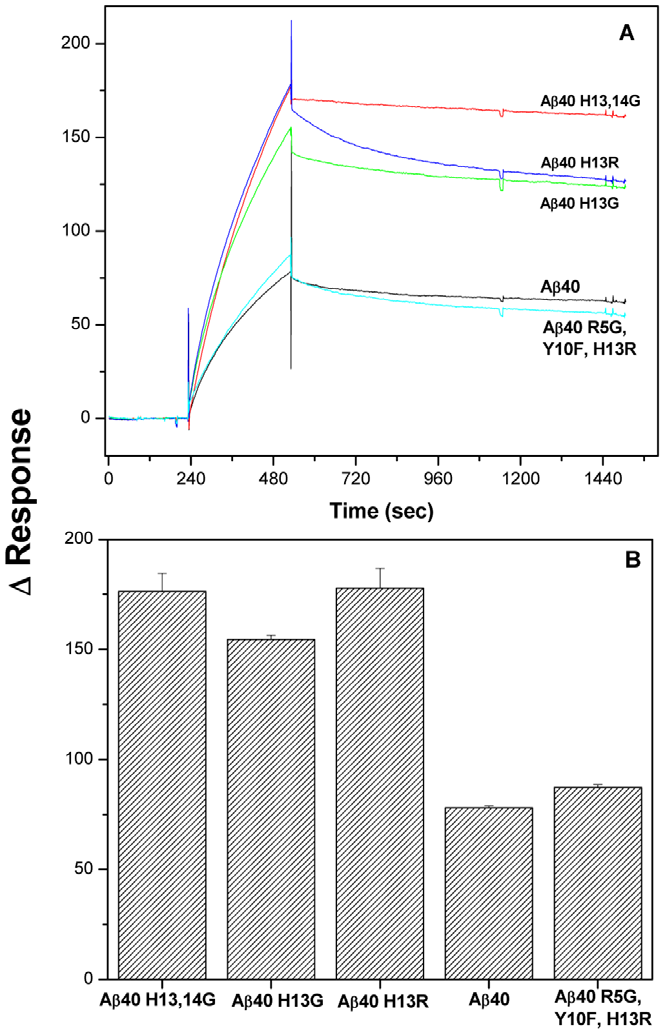
Substitution of the histidine residues of Aβ increases its avidity to human Aβ40 fibrils compared to the native or rodent Aβ as demonstrated by surface plasmon resonance. (A) Freshly prepared Aβ40 derivatives (75 µM) were injected over immobilized Aβ40 fibrils at a flow rate of 30 µl/min for 5 min and the dissociation monitored for 15 min. Reference subtracted sensograms are overlayed. (B) Bar chart shows the difference in relative response units of different Aβ derivatives binding to Aβ40 fibrils at the end of injection time (Time = 525 sec).

The binding of various Aβ derivatives to immobilized Aβ40 fibrils was studied using SPR technique. Freshly prepared monomeric peptides were injected at a concentration of 75 µM across an immobilized Aβ40 fibril surface. Prior concentration studies found that 75 µM concentration provided optimized sensograms for all proteins. Figure 4A shows specific binding of various Aβ peptide derivatives to Aβ40 fibrils. The peptides modified at the histidine positions showed nearly a two fold enhanced binding response compared to that of native human Aβ40 or rodent Aβ40 (Aβ40 R5G, Y10F, H13R) (Figure 4B). The kinetic parameters shown in the Table 2 were obtained by fitting the association and dissociation phases simultaneously to a Langmuir model provided with the Biaevaluation software. The goodness of fit was confirmed based on the low chi-square (χ^2^) error values and visual examination of the residual error distribution. The kinetic parameter values provided in Table 2 were determined to be highly significant using Student's t-test.

**Table 2:** Kinetic Rate Constants of Aβ40 Derivatives Binding to Immobilized Aβ40 Fibrils Determined by Surface Plasmon Resonance.

Peptide	k_a_ (M^-1^s^-1^)×10^2^	k_d_ (s^-1^) ×10^-4^	K_D_ (M) ×10^-6^	χ^2^
Aβ40 H13, 14G	0.45	0.5	1.2	0.75
Aβ40 H13R	0.39	2.9	7.5	1.25
Aβ40 H13G	0.51	1.3	2.5	4.35
Aβ40	0.55	1.6	2.9	0.99
Aβ40 R5G, Y10F, H13R	0.31	3.3	10.8	1.47

The association rate constant (k_a_) of various Aβ derivatives binding to fibrillar Aβ40 varied between 31 to 55 M^-1^s^-1^. Aβ40 exhibited the maximum k_a_ value of 55 M^-1^s^-1^ followed by Aβ40H13G, Aβ40H13,14G, Aβ40H13R, and the rodent Aβ40 R5G, Y10F, H13R. Statistically significant differences were also observed between the dissociation rate constants (k_d_) of various peptides from the fibrillar Aβ40. Aβ40H13,14G demonstrated the slowest dissociation rate followed by Aβ40H13G, Aβ40, Aβ40H13R, and the rodent Aβ40 R5G, Y10F, H13R. The equilibrium binding constant (K_D_), which has an inverse relationship to the affinity of various peptides to the Aβ40 fibrils, was the highest for rodent Aβ40 R5G, Y10F, H13R followed by Aβ40H13R, Aβ40, Aβ40H13G, and Aβ40 H13, 14G. These results demonstrate that Aβ40H13, 14G has a higher affinity to Aβ40 fibrils with low equilibrium dissociation constant of 1.2 µM compared to the rest of the peptides (Figure 4).

The results from SPR studies demonstrate that whether Aβ40 is in a monomeric or randomly oriented structure, removal of the imidazole side chain from histidine at position 13 and 14 results in increased affinity when it interacts with the β-sheet Aβ40 fibrils. This histidine substitution results in a strong interaction with fibrillar Aβ40 and takes more time to dissociate from the fibrillar surface. The histidine modification at positions 13 and 14 of Aβ40, therefore, increases its avidity to fibrillar Aβ40.

### 3.5 Labeling of Amyloid Plaques in APP, PS1 Mouse Brain Sections In Vitro with Radioiodinated Aβ40, Aβ40 H13G, Aβ40 H13R, or Aβ40 H13,14G

**Figure 5:**
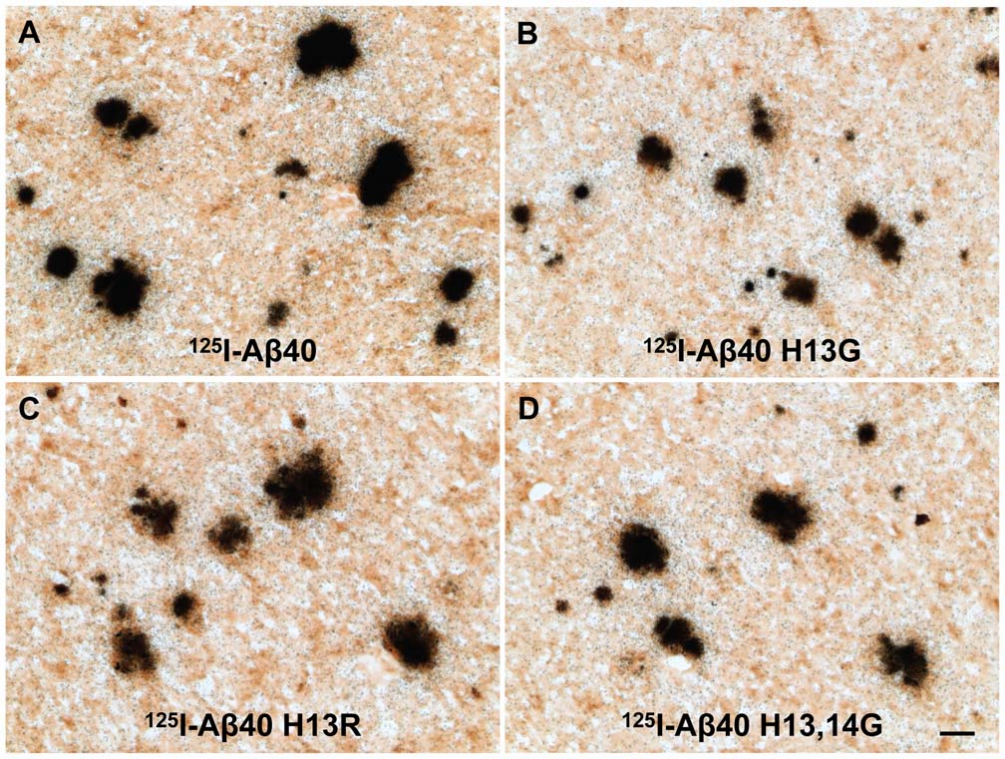
Labeling of amyloid plaques in APP, PS1 mouse brain sections in vitro with radioiodinated Aβ40 (A), Aβ40 H13G (B), Aβ40 H13R (C), or Aβ40 H13,14G (D). HPLC-purified ^125^I-Aβ40, ^125^I-Aβ40 H13G, ^125^I-Aβ40 H13R, or ^125^I-Aβ40 H13,14G were incubated *in vitro* with unfixed cryosections (15 µm) of brain from a 20-month old APP, PS1 AD transgenic mouse. Sections underwent anti-amyloid IH and emulsion microautoradiography and were developed after one day of exposure. Scale bar equals 50 µm.

HPLC-purified ^125^I-Aβ40, ^125^I-Aβ40 H13G, ^125^I-Aβ40 H13R, and ^125^I-Aβ40 H13,14G were incubated *in vitro* with unfixed cryosections of brain from a 20-month old APP, PS1 AD transgenic mouse. The results are illustrated in Figure 5, which displays micrographs of typical binding in mouse cortex for each peptide. All four peptides labeled amyloid plaques equally well, and in all regions of mouse brain, exposing many silver grains after only one day of exposure. These binding studies were performed twice in mouse brain sections and once in human AD brain sections with the same results. With equal counts or amounts of peptide, all four Aβ40 peptides labeled plaques with more affinity than any of the Aβ30 derivatives that were tested previously [37].

## Discussion

**Figure 6:**
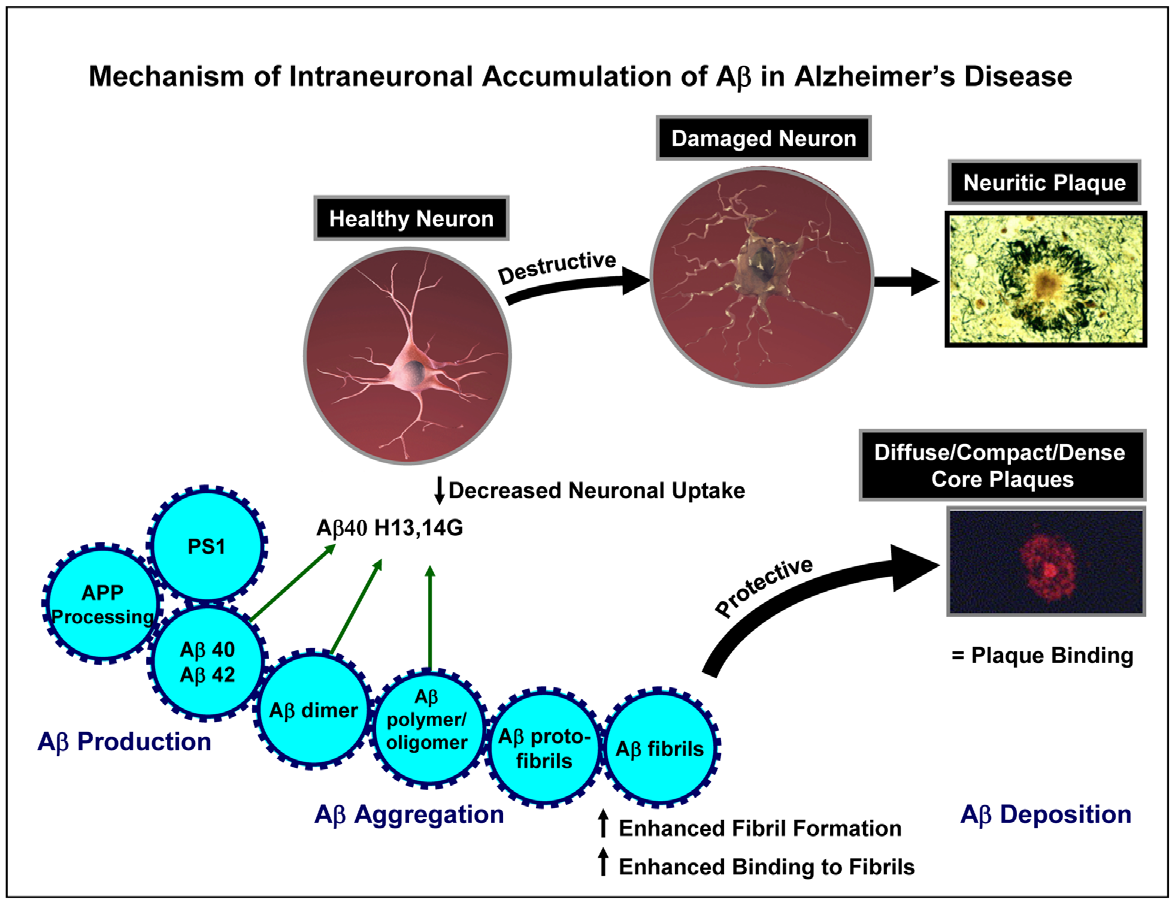
Suggested mechanism of intraneuronal accumulation of Aβ in Alzheimer’s disease. Two pathways for the production of amyloid plaques are displayed. One involves the formation of neuritic plaques, which are characterized by the presence of densely packed Aβ fibrils, dystrophic neurites, reactive gliosis (astrocytes), extracellular filaments, activated microglial, and numerous other proteins. It is hypothesized that this neuritic plaque results from increased neuronal binding of Aβ, which forms fibrils that result in damaged neurons that follow a destructive pathway ultimately leading to the formation of the neuritic plaque. In contrast, Aβ that is not taken up by neurons forms Aβ protofibrils and fibrils and follows a protective pathway that results in the formation of diffuse/compact/dense core plaques. Substitution of the histidines of Aβ40 at positions 13 and 14 with glycine results in a significant decrease in neuronal binding, enhanced fibril formation, enhanced binding to fibrils, and equivalent plaque binding and thereby driving the histidine-substituted Aβ to the protective pathway involving the formation of diffuse/compact/dense core plaques. The plaque images are taken with permission from [45] and [46].

These data suggest that the mechanism of neuronal binding of Aβ in Alzheimer’s disease involves the adjacent histidine residues of Aβ which facilitates a biophysical interaction with neuronal cell surface lipids and proteins that results in its accumulation in healthy neuronal cell bodies and dendrites (Figure 6). It is likely that the intraneuronal Aβ forms fibrils, which ultimately affects a variety of functions within the neurons characterized by alterations in synaptic function and the onset of dementia. These damaged neurons eventually die and lead to the production of neuritic plaques which are characterized by accumulation of Aβ fibrils, dystrophic neurites, reactive gliosis (astrocytes), extracellular filaments, and activated microglial, as well as a host of other proteins that have been found associated with these neuritic plaques. We hypothesize that this is a destructive pathway that occurs over a long period of time (perhaps decades) in Alzheimer’s disease. In contrast, a large amount of Aβ that is produced forms soluble monomers, dimers, and oligomers and ultimately insoluble protofibrils and fibrils that result in the Aβ deposition as diffuse/compact/dense core plaques. We suggest that this is a protective mechanism for removing excessive production of Aβ. Interestingly, those Aβ deposits that are found in brains of pathological aging individuals without clinical symptoms of cognitive deficits are exclusively diffuse amyloid plaques and support this proposed protective mechanism for removing excess levels of Aβ.

Several mechanisms have been proposed to describe the interactions of soluble Aβ proteins with neurons which ultimately cause neurodegeneration. In a recent report, Simakova and Arispe [38] have shown that Aβ proteins elicit their toxicity by binding to the neuronal cell membrane. On the other hand, numerous reports have claimed that Aβ proteins are internalized by neurons via receptor mediated endocytosis, escape degradation in lysosomes, and accumulate in the cytosol [39]-[41]. We have recently proposed a radically different mechanism that Aβ proteins are internalized by neurons via non-endocytotic and energy independent pathways, most likely due to their ability to biophysically interact with the neuronal membrane [13]. Consequently, a significant amount of the internalized protein accumulates outside of the endosomal or lysosomal compartments in the neuroplasm without degradation. Whether the neurons are distressed by mere binding of Aβ proteins to the neuronal membrane or by internalization of the Aβ proteins that trigger cellular events that eventually lead to synaptic loss and neurodegeneration needs further resolution. Nevertheless, both situations are impacted by the ability of Aβ proteins to bind to the neuronal membrane. It would be important to further assess neuronal binding of Aβ42 as well as oligomers of Aβ. The assessment of oligomeric binding might be more problematic as the fluorescein derivative is added to Aβ during its synthesis which might affect subsequent formation of oligomers.

Recent studies have investigated Aβ-cell membrane interactions which were found to be inhibited by annexin V, a specific ligand for phosphatidylserine [42], [43]. Membrane binding of Aβ that is required for its cell-selective neurotoxicity was also shown to be determined by surface phosphatidylserine [38]. It was recently shown that annexin V inhibited L-Aβ42 but not D-Aβ42 binding to cultured cortical neurons yet rescued L-Aβ42 neurotoxicity [44], thus emphasizing the importance of phosphatidylserine in the biophysical binding of Aβ to neurons.

The current study provides critical information on the amino acids that enable Aβ peptide to anchor to the neuronal membrane. The experiments reported in this study demonstrated that substitutions of the adjacent histidines residues of fluoroscein-labeled Aβ40 (F-Aβ) with glycine decreased binding/uptake in differentiated PC12 cells by approximately 60% as assessed by flow cytometry analysis (Figure 1). However, no significant differences were observed in cells treated with trypsin, which removed most of the peptides bound to the cell surface. More interestingly, there was no difference in F-Aβ40 H13,14G fluorescence before and after trypsin treament suggesting that it’s binding to PC12 cells is very modest. It is obvious from this data that F-Aβ40 has higher binding to PC12 cells than F-Aβ40 H13,14G. The shorter incubation times (20 min) used for these studies are appropriate to reach conclusions on the binding, but they are very short to draw conclusions on the cellular uptake behavior. Studies have to be conducted with longer incubation times to compare the extent of internalization of these peptides in neuronal cells. To more accurately reflect the situation in normal brain, the binding of these peptides to adult neurons was also determined in a mouse brain slice model. The H13,14G substitutions on F-Aβ40 substantially reduced binding to cortical neurons by 65% and to hippocampal neurons by 52% in WT brain slices compared to F-Aβ40 (Figure 2). Clearly, the adjacent histidine residues on Aβ40 play an important role in at least some of the binding of Aβ40 to the neuronal surface and probably for its internalization as well.

These adjacent histidine substitutions on Aβ40 also enhanced fibril formation as evaluated by ThT binding (Figure 3) and increased binding to Aβ fibrils as demonstrated by surface plasmon resonance (Figure 4), with a sustained ability to bind to amyloid plaques (Figure 5). Substitution of the histidine residues, therefore, is hypothesized to drive Aβ along a protective pathway that will ultimately result in the increased accumulation of diffuse/compact/dense core plaques (modeled in Figure 6). Substitution of these histidine residues or agents that would mask these histidine residues may have therapeutic potential for preventing neuronal uptake and subsequent accumulation in neurons and ultimately the destruction of the neuron and drive the protein to the protective pathway involving diffuse/compact/dense core plaque accumulation.

Because of the presumed protective role of diffuse/compact/dense core plaques, which are formed from the nuclei of Aβ fibrils, the propensity of various Aβ derivatives to form fibrils was investigated. The kinetics of fibril formation was primarily followed using a ThT-binding assay (Figure 3). The H13R modification, which substitutes positively charged histidine with arginine of the same charge on Aβ40, increased the rate of fibril formation and the lag time slightly. Rodent Aβ40 that harbors H13R modification along with two other modifications (R5G, and Y10F), also demonstrated similar fibril formation kinetics. However, when the histidine at position 13 was substituted with an amino acid carrying no charge at physiological pH, such as glycine, the rate of fibril formation increased significantly; however, the lag time also increased. Substituting histidines at both positions 13 and 14 with glycine enhanced the rate of fibril formation further and also extended the lag time significantly.

To analyze these interesting trends further, the kinetic data was fitted to a model that describes Finke-Watzsky mechanisms of nucleation followed by autocatalytic surface growth. The curve fitting results outlined in Table 1 demonstrated that the nucleation rate of Aβ40 was much greater than any of the derivatives. However, the nucleation rate (k_1_) decreased drastically (1/10000 to 1/1000000) followed by a modest 3-fold increase in the rate of fibril growth (k_2_) for the peptides with H13R substitution (Aβ40 H13R and rodent Aβ40), which was most likely responsible for the observed lag times. In the case of H13 substitution to glycine, the rate constants k_1_ and k_2_ decreased to 1/9 and 2/3 compared to that of native Aβ40. These changes in k_1_ and k_2_ can explain the prolongation of lag time with Aβ40 H13G compared to Aβ40, but fall short of explaining why this substitution forms more fibrils than the native Aβ40. The magnitude of these kinetic trends increases remarkably with the substitutions of both adjacent histidines at positions 13 and 14 with glycines, suggesting that the fibril formation kinetics of Aβ40 H13G and Aβ40 H13,14G deviate from the classical Finke-Watzsky mechanism. This view point is also supported by the observation that the model did not fit the nucleation and fibril growth phases of Aβ40 H13G and Aβ40 H13,14G as well as it did with the other derivatives. Although the Finke-Watzsky model is the simplest model capable of describing various factors that characterize nucleated aggregation of various neurological proteins, including Aβ40, this phenomenological model may be inadequate, (also acknowledged by Morris et. al [36]), in describing the events of a complex multistep aggregation process. More detailed biophysical studies are being conducted in our lab to study the unique aggregation mechanism of the Aβ40 H13G and Aβ40 H13,14G peptides.

The AD brain is expected to contain a significant amount of pre-existing Aβ fibrils and diffuse plaques. The Aβ40 derivatives are expected to bind to these amyloid forms, thus making them unavailable for neuronal interactions. Therefore, the ability of Aβ40 derivatives to bind to preformed Aβ fibrils and to amyloid plaques in the APP, PS1 mouse brain sections was determined using surface plasmon resonance (SPR) and emulsion autoradiography techniques, respectively. The SPR studies demonstrated that Aβ40 H13,14G maintains the highest binding to preformed Aβ fibrils (Figure 4). Moreover, this derivative also exhibited similar binding to amyloid plaques in the AD mouse brain as Aβ40 (Figure 5). Hence, masking of these histidine residues may drive Aβ toward protective plaque formation rather than neuronal binding and accumulation.

In summary, these experiments indicate that the HH domain of Aβ provides at least in part the structural basis for its neuronal binding.
